# Are changes in perceived physical workload and strenuousness of work among partial disability pensioners associated with full disability pension?

**DOI:** 10.1177/14034948251326706

**Published:** 2025-03-28

**Authors:** Mari-Anne Wallius, Anne Kouvonen, Jenni Ervasti, Taina Leinonen, Jaakko Airaksinen, Tea Lallukka

**Affiliations:** 1 Finnish Institute of Occupational Health, Helsinki, Finland; 2 Faculty of Social Sciences, University of Helsinki, Helsinki, Finland; 3 Centre for Public Health, Queen’s University Belfast, Northern Ireland; 4 Department of Public Health, University of Helsinki, Helsinki, Finland

**Keywords:** Workload, physical work, disability pension, partial disability pension, retirement, aged, cohort studies, registries, longitudinal studies

## Abstract

**Aims:**

The aim of this study was to examine the changes in pensioners’ perceived physical workload and the physical strenuousness of work before and after transition to partial disability pension (pDP), and whether these changes were associated with subsequent transition to full disability pension (fDP).

**Methods:**

We used survey data on physical workload and the physical strenuousness of work and covariates from the Helsinki Health Study, an occupational cohort measured in four phases: 2000–2002, 2007, 2012 and 2017. These data were prospectively linked to the Finnish Centre for Pensions’ register data on pDP and fDP up to 2021 for those who had provided informed consent for such linkage (mean follow-up of 3 years). We included those who responded to the study surveys both before and after pDP (*n* = 235). Changes after transition to pDP in the physical strenuousness of work and in a constructed physical workload summary scale were analysed using log-binomial regression.

**Results:**

After transition to pDP, physical workload decreased among 20% of the participants, remained constantly low among 44% and constantly high among 36%. The changes in the physical strenuousness of work were similar. Thirty per cent (*n* = 70) of pDPs transitioned to fDP during the follow-up. The changes in physical workload or the physical strenuousness of work were not associated with transition to fDP.

**Conclusions:**

**Despite the transition to pDP and thus part-time work, perceived physical workload and the physical strenuousness of work remained stable, and were not reflected in transition to fDP. More research on larger samples is warranted.**

## Introduction

Populations are rapidly ageing across the globe [1]. Therefore, the employment rates of people with reduced work ability must be improved [2]. Utilising everyone’s remaining work capacity is desirable from the perceptive of the individual, the availability of labour and the sustainability of public finances. Many countries provide partial disability benefits to individuals with disabilities, to help them remain in employment. In some European countries such as Sweden, Norway, the Netherlands and Switzerland, disability benefits are frequently based on the level and duration of disability as well as the capacity to work or earn [3]. In Finland, partial disability pension (pDP) is a benefit that encourages people with partial work ability to remain at work, as it allows part-time work [4, 5].

In Finland, the use of pDP has increased in recent decades [5]. In 2022, approximately 30% of all new disability pensions (DPs) under the earnings-related pension scheme were pDPs [6]. Partial DPs are more common among women, and the majority are granted to people over the age of 55 [6, 7]. In 2022, 49% of all new pDPs were granted due to musculoskeletal disorders and 23% for mental disorders [6].

A Finnish register-based study (*N* = 5277) showed that during a 4-year follow-up, a third of those on pDP transitioned to full disability pension (fDP) [8]. Another study reported that only 2% of those on pDP returned to full-time work during a 6-year follow-up [4]. Higher age, male gender and working in the public sector increased the risk of transitioning from pDP to fDP [8]. In addition, those on pDP and in a lower occupation-based socioeconomic position [4] and with lower education more often transitioned to fDP [8]. Although this transition is based on ill health, it also depends on several factors related to work. In the general population, high physical workload and ergonomic job exposures have been associated with disability retirement [9–13], particularly due to musculoskeletal disorders [14–16].

Those on pDP are often exposed to physically demanding work [17, 18]. Work in physically demanding occupations, assessed at an aggregate level on the basis of the job exposure matrix, has remained relatively stable over time both before and after the start of pDP and has not been associated with subsequent fDP [17]. Uncomfortable working postures in the work of people on pDP have nevertheless been associated with an increased risk of transitioning to fDP [18]. However, we are not aware of any studies of changes in physical work demands in the jobs of those on pDP and their associations with transitioning to fDP. That is, it is unclear how individuals whose working hours have been reduced due to their pDP perceive the amount of physical effort (i.e., physical workload factors) and the intensity of effort (i.e., physical strenuousness of work) required in their job before and after their transition to pDP, and how any potential changes in these physical work demands, are related to subsequent transition to fDP. Previous studies of the general population have found that constantly heavy or increased physical workloads are associated with fDP [12] and that changing to an occupation with lower exposure to physical workload reduces the risk of fDP [19]. In Finland, those on pDP generally continue in jobs with similar exposures to those before their transition to pDP [17].

To be able to efficiently support the remaining work ability of those on pDP and to prevent them from exiting the workforce, we need to better understand how work-related factors contribute to transition to fDP. Our aim was to examine whether physical workload and the physical strenuousness of work change after the transition to pDP and whether these changes are associated with transition to fDP.

## Methods

**Figure 1.**
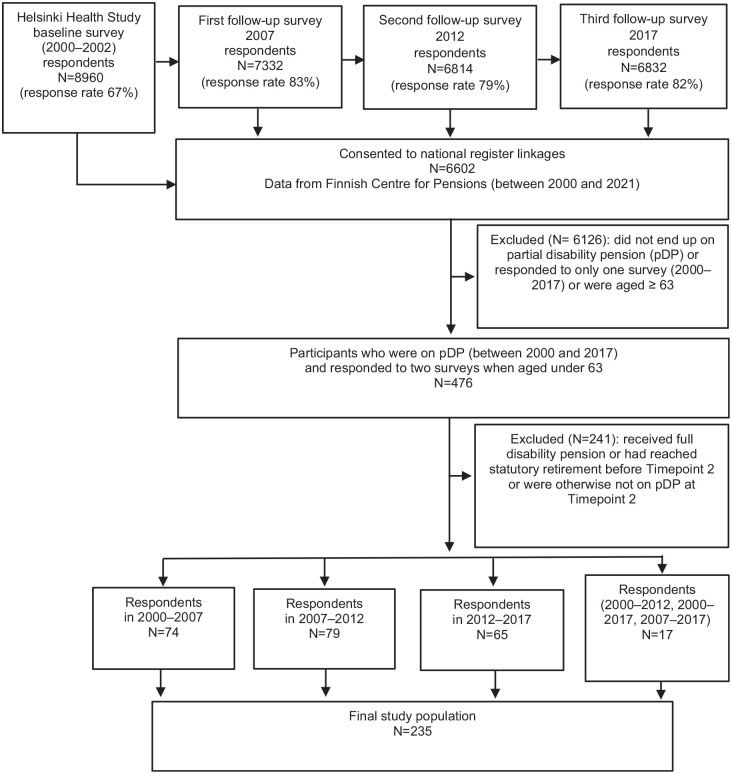
Flow chart of study population selection. Participants responded to surveys both before (Timepoint 1) and after (Timepoint 2) being granted partial disability pension (pDP). If the participant had more than two survey responses, the first survey during which the participant was on pDP was selected as Timepoint 2 (either 2007, 2012 or 2017) and the one prior to this as Timepoint 1 (either 2000–2002, 2007 or 2012).

We used survey data from the Helsinki Health Study, which examines the health and well-being of employees of the City of Helsinki, Finland. Employees turning 40, 45, 50, 55 and 60 in 2000, 2001 and 2002 (*n* = 8960, response rate 67%) responded to the baseline survey and then to follow-up surveys in 2007, 2012 and 2017 (Figure 1). Further details on the cohort can be found elsewhere [20].

We linked the responses to the surveys (Phases 1–4 collected in 2000–2002, 2007, 2012 and 2017, respectively) to individual-level data obtained from the registers of the Finnish Centre for Pensions from 2000 until the end of 2021 for those who had provided written consent for such linkage (*n* = 6602, 74%). All the participants were employed during Phase 1. For the purposes of this study, we focused on the participants who responded to the surveys both before (Timepoint 1) and after (Timepoint 2) who had been granted pDP and were aged below 63 (fDP can no longer be granted at the age of 63). Thus, Timepoint 2 was either 2007, 2012 or 2017 and Timepoint 1 was prior to this (either 2000–2002, 2007 or 2012, respectively). A total of 476 participants were on pDP between 2000 and 2017 and responded to two surveys when aged below 63 (Figure 1). We excluded those who received fDP or began statutory retirement before Timepoint 2 or were otherwise not on pDP at Timepoint 2 (*n* = 241). The final study population was 235 participants.

### Physical workload and physical strenuousness of work

Physical workload factors were measured at each survey phase using an 18-item instrument developed at the Finnish Institute of Occupational Health [21]. Each item had four response alternatives: (i) does not occur, (ii) occurs, but does not bother me at all, (iii) occurs and somewhat bothers me, (iv) occurs and bothers me a lot. Previous studies [11, 22, 23] using factor analysis have found three broad work exposure factors, one of which has been deemed best for measuring physical workload. We used the six items that in these previous studies loaded on the physical workload factor: (i) uncomfortable working postures, (ii) repetitive rotation of the trunk, (iii) repetitive movements, (iv) heavy physical effort or lifting and carrying heavy loads, (v) walking and (vi) standing. We constructed a summary scale of physical workload based on these six physical workload factor items (Cronbach’s alpha, α = 0.85), and dichotomised the score from the median. Changes in physical workload were measured using a four-category variable of change for the summary scale: (i) constantly low physical workload (none/low at Timepoint 1 and Timepoint 2), (ii) increased physical workload (none/low at Timepoint 1 and high at Timepoint 2), (iii), decreased physical workload (high at Timepoint 1 and none/low at Timepoint 2), and (iv) constantly high physical workload (high at both Timepoint 1 and Timepoint 2).

We also assessed the physical strenuousness of work using a single-item question with four response options (‘very light’, ‘rather light’, ‘rather strenuous’ and ‘very strenuous’), eliciting how physically strenuous the respondent considered their work. We dichotomised the strenuousness of work into physically non-strenuous and physically strenuous, and further classified it into a four-category variable of change (constantly non-strenuous, increased strenuousness, decreased strenuousness and constantly strenuous). None of the participants’ physical strenuousness of work or physical workload increased, so we used only the three remaining categories in the analyses.

### Disability pension

We obtained register-based information on all granted DPs and statutory pensions from the Finnish Centre for Pensions, which has records on all pension recipients from the earnings-related pension scheme. According to this scheme, pDP and fDP can be granted either temporarily or permanently to Finnish residents aged 18–62 with a work history that has accrued a pension [24]. DP can be granted in partial form to an employee who has lost at least 40% of their work ability due to illness for at least 1 year. This means that the degree of the individual’s work disability has to be at least 40%. If it is at least 60%, the individual can apply for fDP [24]. The amount of the pDP benefit is half of the full benefit, which is the sum of the individual’s pension that had accrued before they became disabled and the projected pension component [24]. Most individuals on pDP work regularly, averaging just over 20 h a week [25]. We focused on all-cause DPs, and pooled temporary and permanent pensions for the analyses. For descriptive purposes, we nevertheless used information on whether the pDP and fDP were granted due to musculoskeletal diseases, mental disorders, or other causes.

### Covariates

We used the data on age, gender (men/women) and education from Phase 1. We classified age into two categories from the median (<45 and ⩾45 years). Education was divided into three levels: (i) basic, (ii) intermediate and (iii) higher education. We considered body mass index (BMI) a covariate because BMI is associated with disability retirement [26]. We calculated BMI as weight in kilogrammes divided by height in metres squared, using self-reports. BMI was measured at both timepoints and was used as a continuous time-variant covariate. Follow-up time was used as a time-variant covariate. The follow-up time for fDP started from the date of the survey at Timepoint 2 (2007, 2012 or 2017) until the date when fDP was granted, statutory pension was granted, the participant turned 63, or the end of the follow-up on 31 December of that particular year, whichever occurred first.

The study was conducted in line with the Helsinki Declaration. Ethics approval was provided by the Ethics Committee of the Department of Public Health, University of Helsinki and the necessary research permits were granted by the authorities of the City of Helsinki, Finland.

### Statistical analyses

We evaluated the proportional hazards assumption graphically from Kaplan–Meier curves and deemed that it did not fulfil the assumption. We used relative risk (RR) estimation with confidence intervals (95% CIs) using log-binomial regression to examine whether the changes in physical workload between Timepoints 1 and 2 were associated with transition to fDP. Similarly, we examined the association between changes in physical strenuousness of work and fDP.

We constructed three models. In Model 1, we analysed the RRs and 95% CIs of the fDP connected with changes in physical workload or the physical strenuousness of work, adjusting for follow-up time, gender and age. Model 2 was adjusted for the same covariates as those in Model 1 plus education. Model 3 was adjusted for all the covariates in Model 2 plus BMI. All data analyses were conducted using IBM SPSS version 27.0.

We performed a sensitivity analysis using the summary scale of change in physical workload as a continuous variable in the model. The change in the sum score was calculated as the change between the two timepoints by subtracting the Timepoint 2 score from the Timepoint 1 score. Models with physical strenuousness of work as the explanatory variable were also adjusted for time on pDP as a covariate by calculating the time between when pDP started and Timepoint 2. Due to missing values in the data, we had 200 observations for physical workload and 215 observations for physical strenuousness of work.

## Results

Table I presents the descriptive characteristics of the study population (*n* = 235). The mean age was 47 (SD 4) and 92% were women. Of the pDP diagnoses, 53% were due to musculoskeletal diseases, 19% due to mental disorders, and the remainder due to other causes.

**Table I.** Characteristics of study population comprising individuals who have transitioned to partial disability pension (pDP). Helsinki Health Study survey (collected in 2000–02, 2007, 2012 and 2017) was linked to register data of the Finnish Centre for Pensions from 2000 to the end of 2021. Study participants responded to the surveys both before (Timepoint 1) and after (Timepoint 2) being granted pDP (*n* = 235).

Characteristics at baseline (before pDP)	All N* (%)	
Gender		
Men	19 (8.1)	
Women	216 (91.9)	
Age (mean, SD)	47.0 (4.1)	
Education		
Basic	109 (46.4)	
Intermediate	89 (37.9)	
High	37 (15.7)	
Characteristics before and after pDP	Timepoint 1 n* (%)	Timepoint 2 n* (%)
Body mass index (mean, SD)	27.6 (5.1)	28.5 (5.4)
Physical strenuousness of work^a^		
Strenuous	106 (49.3)	104 (48.4)
Non-strenuous	109 (50.7)	111 (51.6)
Physical workload (sum variable),^b^ mean (SD)	14.8 (4.4)	14.6 (4.6)

* Numbers vary because some survey responses were missing.

a Strenuousness of work assessed by question on how physically strenuous the respondent considered their work and responses were dichotomised into physically non-strenuous (‘very light’ and ‘rather light’) and physically strenuous (‘rather strenuous’ and ‘very strenuous’).

b Summary scale of physical workload based on six physical workload items (further categorised as a four-category variable of change in physical workload using median as cut-off point in the analysis).

After transitioning to pDP, physical workload decreased among 20% of the participants, and remained constantly low among 44% and constantly high among 36%. Those with pDP due to musculoskeletal diseases had the largest proportion of constantly low (56%) and those with mental disorders constantly high (58%) physical workload. Physical workload did not increase from low to high for any of the participants.

Of the 235 participants who transitioned to pDP, 70 (30%) ended up on fDP during the maximum 14-year follow-up period. The diagnosis group distribution of the fDPs resembled that of the pDPs. Changes in physical workload were not associated with transition to fDP (Table II). After adjustment for covariates, the estimated RR for the decreased physical workload group was 0.74 (95% CI 0.39–1.41) and for the constantly high workload group, 0.98 (95% CI 0.58–1.67). Sensitivity analyses with a continuous summary scale of change in physical workload showed no association with transition to fDP (RR 1.06, 95% CI 0.99–1.14 for full model).

**Table II.** Relative risks (RRs) and their 95% confidence intervals (CIs) for associations between changes in physical workload and physical strenuousness of work from Timepoint 1 to Timepoint 2 and subsequent full disability pension in a maximum 14-year follow-up. Participants responded to the surveys before (Timepoint 1) and after (Timepoint 2) being granted partial disability pension (total *n* = 235, *n* of events = 70). Helsinki Health Study survey carried out in 2000–02, 2007, 2012 and 2017. Register data of Finnish Centre for Pensions from 2000 to the end of 2021.

		Changes in physical workload* (*n* = 200)		
		Constantly low (*n* = 88)	Constantly high (*n* = 73) RR (95% CI)	Decreased (*n* = 39) RR (95% CI)
Model	*N* of events^#^	28	19	9
Model 1^a^	56	1.0	0.92 (0.56–1.52)	0.71 (0.37–1.34)
Model 2^b^	56	1.0	0.99 (0.58–1.67)	0.74 (0.39–1.42)
Model 3^c^	55	1.0	0.98 (0.58–1.67)	0.74 (0.39–1.41)
		Changes in physical strenuousness of work** (*n* = 215)		
		Constantly non-strenuous (*n* = 94)	Constantly strenuous (*n* = 89) RR (95% CI)	Decreased (*n* = 32) RR (95% CI)
Model	*N* of events^#^	27	27	7
Model 1^a^	61	1.0	0.99 (0.64–1.53)	0.69 (0.33–1.43)
Model 2^b^	61	1.0	0.96 (0.60–1.54)	0.72 (0.35–1.50)
Model 3^c^	60	1.0	0.98 (0.62–1.57)	0.72 (0.34–1.51)

* Physical workload between two timepoints measured with constructed summary scale of physical workload. A high score indicates high exposure. Physical workload summary scale was dichotomised from the median and categorised as a four-category variable of change in physical workload. Physical workload did not change from low to high for any of the participants. The three categories were: constantly low, constantly high and decreased physical workload.

** Physical strenuousness of work assessed by question on how physically strenuous participants considered their work and responses were dichotomised into physically non-strenuous (‘very light’ and ‘rather light’) and physically strenuous (‘rather strenuous’ and ‘very strenuous’). Change in strenuousness of work classified as four-category variable of change (constantly non-strenuous, constantly strenuous, decreased from strenuous to non-strenuous or increased from non-strenuous to strenuous). The strenuousness of work did not change from non-strenuous to strenuous for any of the participants.

# Numbers vary because some survey responses were missing.

a Change in physical workload or physical strenuousness of work, adjusted for follow-up time, gender and age.

b Change in physical workload or physical strenuousness of work, adjusted for follow-up time, gender, age and education.

c Change in physical workload or physical strenuousness of work, adjusted for follow-up time, gender, age, education and body mass index (BMI).

The physical strenuousness of work did not increase for any of the participants. The physical strenuousness of work decreased among 15% of the participants, remained constantly non-strenuous among 44% and remained constantly strenuous among 41%.

Changes in the physical strenuousness of work were not associated with transition to fDP (RR 0.72, 95% CI 0.34–1.51 in the decreased strenuousness group; and RR 0.98, 95% CI 0.62–1.57 in the constantly strenuous group), after adjustment for covariates (Table II). According to the sensitivity analyses, the average time spent on pDP before the second survey (Timepoint 2) was 2.1 years (SD 1.7), and changes in the physical strenuousness of work were not associated with transition to fDP (RR 0.66, 95% CI 0.31–1.42 in the decreased strenuousness group; and RR 0.91, 95% CI 0.56–1.47 in the constantly strenuous group) in the full model.

## Discussion

This study investigated how perceived physical workload and the physical strenuousness of work changed after transition to pDP, and how these changes were associated with transition to fDP. The main finding was that for the majority of those on pDP, physical workload and the physical strenuousness of work remained at the same level as that before pDP. For 20%, we observed a decrease in physical workload, and correspondingly, 15% reported a decrease in the physical strenuousness of work. Physical workload or the strenuousness of work did not increase for any of the participants on pDP. Changes in physical workload or the physical strenuousness of work were not associated with transition to fDP.

The reason that no one reported an increased physical workload or strenuousness of work might have been reduced working hours. In general, it has been suggested that a reduction in working hours decreases workload and is also beneficial for enhancing recovery [27]. However, physical workload changed from high to low for only a fifth of those transitioning to pDP. One possible explanation for this might be that those on pDPs who worked full working days still perceived their physical workload as high even if they worked only 2 or 3 days a week. Age-related decline in physical capacity may also have contributed to the individuals’ perception that their workload had not reduced. In addition, changes to work tasks may potentially have influenced how they assessed their workload. Furthermore, 44% of the participants had a low physical workload both before and after pDP, meaning that their physical workload could no longer decrease from baseline. This, in turn, relates to the fact that more than half of the participants had either an intermediate or high education level, and the majority of those with a high education level reported physically non-strenuous work both before and after pDP. Earlier studies have indicated that most of those who start receiving pDP continue to work in the same occupation or in a similar position [17, 25], and our findings support this observation. However, we cannot rule out the possibility that we may have overlooked some changes in physical workload, as we used the median as the cut-off point for the physical workload change.

Although stable high or increased physical workload has been related to disability retirement in the general working population [12], we found no association between changes in physical workload (or the strenuousness of work) and transition to fDP among those on pDP. However, given the small size of the study population we cannot firmly conclude that these links do not exist.

Our results contribute to prior research by providing new evidence of changes in physical workload and the strenuousness of work among those transitioning to pDP. Our findings indicated that 41% had physically strenuous work both before and after being granted pDP. Such evidence is important for understanding how individuals with partial work disability perceive the strenuousness of their work after a reduction in working hours and for determining whether their physical working conditions need any further modifications. We lacked information on occupation, which is associated with the presence or absence of adverse physical working conditions. Occupation nevertheless correlates highly with education, which we included in the analyses as a covariate.

A strength of this study was its complete national register data with no loss to follow-up for fDP among the study participants. The data on physical workload and the physical strenuousness of work were obtained through self-reports rather than by direct measurement of working conditions. Thus, we gained valuable information on the experiences of those on pDP and supplemented previous research. The self-reports of those on pDP regarding the physical strenuousness of their work were also in line with the subjective perceptions of changes in physical workload. It is noteworthy, however, that individuals with poor health have been shown to be more likely to report a high physical workload [28]. The poor health of individuals on pDP may have therefore led to biased self-reports, resulting in over-reporting of high physical workload.

One limitation of the study is that the changes in physical workload and strenuousness of work were defined on the basis of two surveys carried out at about 5-year intervals. This is rather a long period for defining these changes and we were unable to identify whether the workload changed back and forth. Although the time on pDP before the second measurement of the strenuousness of work also varied among the participants, our sensitivity analyses showed that this had no effect on the results concerning the association between the physical strenuousness of work and transition to fDP.

The small study population size is a major limitation of the study. Due to the small study population and the low number of events, this study does not encompass all the factors relevant to the DP process. This also limited our ability to carry out separate analyses for women and men, and for different diagnostic groups. We addressed only some of the work-related factors, specifically the physical dimension. We also only considered a limited portion of the sociodemographic dimension (age, gender and education) and health behaviour-related factors (only BMI). We lacked power in our analyses, and any interpretations therefore merit caution. It is also noteworthy that those on pDP perceived more psychosocial stressors at work than those not on pDP [4]. In order to adapt the work of those on pDPs to their needs, it is also important to obtain information on any potential changes in the perceived mental strenuousness of work when transitioning to pDP and its predictive value for full disability. Furthermore, our findings might not be directly generalisable to other countries with different pension systems to that in Finland. However, pDPs might resemble similar benefits to those of other countries if they allowed part-time work in cases of long-term disability. Women constituted the majority of the study population (92%), as in the Finnish municipal sector in general (80%) and among those retiring on pDP in the general population (68%) [6]. In the municipal working population, women more often report a high physical workload than men [20]. Thus, we cannot generalise our results to apply to men with certainty.

In this study, most participants had either a constantly low or a constantly high physical workload. Although physical workload seemed to remain fairly stable when transitioning to pDP, and thus part-time work, we cannot conclude whether this stable low amount of physical effort (as well as decreased physical effort) was solely due to the reduction in working hours or whether it was also because working conditions had improved. The small study population prevented us from drawing strong conclusions about the changes in physical effort and their possible associations with fDP. A replication of this study could use larger samples. Interventions improving working conditions may have the potential to decrease the number of individuals exiting the labour market due to fDP [29]. Workplaces play a crucial role in helping those with partial disabilities to remain at work and in making the necessary arrangements [30]. Occupational health professionals are often involved in workplace adjustments for individuals with disabilities [31]. Further research is needed on the effectiveness of occupational health interventions for helping those on pDPs to remain in work.

## Conclusions

Although these results require further replications with larger samples, the study nonetheless provides important initial insights. Both perceived physical workload and the physical strenuousness of work remained constant for most participants, regardless of transitions to pDP. The changes in physical workload or physical strenuousness of work reported by the minority of the participants were not reflected in transition to fDP. In addition to reduced working hours, workplace adjustments may be needed for those on pDP who find their work physically demanding.
